# Hepatitis C Virus (HCV) Infection May Elicit Neutralizing Antibodies Targeting Epitopes Conserved in All Viral Genotypes

**DOI:** 10.1371/journal.pone.0008254

**Published:** 2009-12-11

**Authors:** Nicasio Mancini, Roberta A. Diotti, Mario Perotti, Giuseppe Sautto, Nicola Clementi, Giovanni Nitti, Arvind H. Patel, Jonathan K. Ball, Massimo Clementi, Roberto Burioni

**Affiliations:** 1 Laboratorio di Microbiologia e Virologia, Università “Vita-Salute” San Raffaele, Milano, Italia; 2 MRC Virology Unit, Institute of Virology, University of Glasgow, Church Street, Glasgow, United Kingdom; 3 Institute of Infection, Immunity and Inflammation, School of Molecular Medical Sciences, University of Nottingham, Queen's Medical Centre, Nottingham, United Kingdom; Pohang University of Science and Technology, Republic of Korea

## Abstract

Anti-hepatitis C virus (HCV) cross-neutralizing human monoclonal antibodies, directed against conserved epitopes on surface E2 glycoprotein, are central tools for understanding virus-host interplay, and for planning strategies for prevention and treatment of this infection. Recently, we developed a research aimed at identifying these antibody specificities. The characteristics of one of these antibodies (Fab e20) were addressed in this study. Firstly, using immunofluorescence and FACS analysis of cells expressing envelope HCV glycoproteins, Fab e20 was able to recognize all HCV genotypes. Secondly, competition assays with a panel of mouse and rat monoclonals, and alanine scanning mutagenesis analyses located the e20 epitope within the CD81 binding site, documenting that three highly conserved HCV/E2 residues (W529, G530 and D535) are critical for e20 binding. Finally, a strong neutralizing activity against HCV pseudoparticles (HCVpp) incorporating envelope glycoproteins of genotypes 1a, 1b, 2a, 2b and 4, and against the cell culture-grown (HCVcc) JFH1 strain, was observed. The data highlight that neutralizing antibodies against HCV epitopes present in all HCV genotypes are elicited during natural infection. Their availability may open new avenues to the understanding of HCV persistence and to the development of strategies for the immune control of this infection.

## Introduction

The hepatitis C virus (HCV) infection is presently a major public health problem, and nearly 3% of the world population is persistently infected with this virus [1], [2]. At least six major HCV genotypes and more than 50 subtypes have been described based on nucleotide diversity of the core, E1\E2, and NS5 regions [3], [4], [5]. Moreover, HCV infection is characterized by high intra-host variability, due to high mutation rate and replication activity in vivo. As a direct consequence of this variability, each HCV-infected patient harbours a population of related, but genetically distinct viral variants. It is presently believed that HCV variability strongly determines virus persistence in the infected hosts, allowing its escape from the immune system [6], [7], [8] .

Despite almost two decades of studies, no vaccine is presently available to prevent infection with this virus. Efforts aimed at developing an anti-HCV vaccine have been hampered by the high virus variability, the substantial lack of readily available animal models, and the absence of clearly established *in vitro* correlates of protective immunity. It is currently believed that CD4+ and CD8+ T-cell responses are central in the control of acute HCV infection. Otherwise, the role of anti-HCV humoral response is still controversial [9], [10], [11]. However, recent data have suggested that neutralizing anti-HCV antibody responses may strongly influence the natural course of HCV infection. In this context, an effective immunization against HCV calls for induction of both robust T- and B-cell responses. As far as the latter point is concerned, this result is possible if new immunogens are designed, capable of eliciting broadly reacting and neutralizing antibodies.

Recently, the availability of viral pseudotypes bearing surface HCV glycoproteins (E1 and E2) has offered a reliable model system to evaluate *in vitro* the anti-HCV neutralizing activity of polyclonal sera and monoclonal antibodies. Using this approach, it has been shown that neutralizing activity is detectable in sera from a significant proportion of infected patients during primary and persistent HCV infection [12], [13], and that the presence of high titres of neutralizing antibodies directed against conserved epitopes may influence the virus-host interplay during all stages of the infection [14], [15], [16], [17], [18]. In the last few years, we have used phage-display to dissect the antibody response of HCV-infected patients, searching for potentially cross-reactive Fabs directed against HCV/E2. Using this approach, we have obtained several cross-reactive anti-HCV/E2 human mAbs endowed with different biological characteristics [19], [20], [21], [22], [23], [24], [25]. In the study shown here, we evaluated the binding characteristics of a human monoclonal Fab (named e20) that was able to bind E2 glycoproteins of all tested genotypes. Notably, this Fab was able to cross-neutralize HCVpp containing E1\E2 from different genotypes.

## Materials and Methods

### Ethics Statement

Previous studies, that allowed the cloning of the human monoclonal antibody used in this research, were approved by the University Vita-Salute Ethical Committee. However, the present study, not presenting experiments on animals, human samples, or patients, and being based on a recombinant protein expressed in a bacterial vector, did not specifically require a formal adjunctive approval.

### Analysis of the Binding Activity

The binding activity of anti-HCV/E2 monoclonal Fab e20 was assayed using recombinant HCV/E2 proteins derived from different genotypes. The following isolates were used: genotype 1a (isolate H77.20); 1b (UKN1B12.16); 2a (UKN2A.2.4); 2b (UKN2B2.8); 3 (UKN3A1.28c); 4 (UKN4.21.16); 5 (UKN5.15.11) and 6 (UKN6.5.8). In brief, human epithelial kidney (HEK) 293T cells were transfected with 3 µg of pcDNA3.1 vector [5], an expression vector encoding surface glycoproteins of the above listed HCV isolates [26]. After centrifugation, fixation and permeabilization, the transfected cells were incubated with e20 (10 µg/ml). After further washings, the cells were incubated with a FITC-conjugated anti-human Fab monoclonal antibody, and analysed by fluorescent microscopy and by FACS. Untransfected cells and a human recombinant Fab (c33-3) specific for a non-structural antigen of HCV (NS3) were also included in each experiment as negative controls [27].

A mouse broadly cross-reactive monoclonal (AP33) was used to analyze the transfection efficiency for each genotype. The percentage of AP33-incubated sorted cells featuring a higher fluorescence signal than untreated cells was comparable for all different genotypes (approximately 70% of 10,000 cells analysed by FACS).

The binding activity of e20 against the different genotypes was then expressed as percentage of the reactivity observed on genotype 1a (relative binding activity), measured by sorting for each genotype the number of cells featuring a higher fluorescence signal than cells without Fab. The fluorescence obtained using e20 on untransfected cells was subtracted as background.

### Epitope Characterization: Competition ELISA

In order to map the e20 epitope, competition ELISA assays were performed using a panel of well-characterized murine and rat monoclonals (Table 1). Competition experiments were conducted reciprocally, as previously described [19], using plates coated with HCV/E2 of genotype 1a (4 µg/ml). In brief, Fab e20 was titrated and used as probe at a concentration giving 50% of maximum binding, whereas all competing mAbs were used at a saturating concentration. In this case, a horse radish peroxidase (HRP)-conjugated anti-human IgG Fab polyclonal was used to detect Fab 20 binding on HCV/E2. Alternatively, each mouse or rat monoclonal was used as probe at a concentration giving 50% of maximum binding, and Fab e20 was used at a saturating concentration. In this case, HRP-conjugated anti-mouse or anti-rat IgG polyclonals were used to detect the binding on HCV/E2. Final results were determined as percent inhibition with the following formula: percent inhibition = 100× [(OD_450_ of probe Fab alone−OD_450_ of probe Fab with competitor Fab)/OD_450_ of probe Fab alone].

**Table 1:** Inhibition of human anti-HCV/E2 Fab e20 binding by competing anti-HCV/E2 rat or mouse mAbs directed against known regions of the antigen.

Competing mAbs	HCV/e2 epitope	e20
**7/59**	384–391	3
**3/11**	412–423	5
**AP33**	412–423	40
**1/39**	436–443	57
**11/20**	436–447	4
**7/16b**	436–447	5
**H47**	452–459	4
**6/1a**	464–471	2
**6/41a**	480–493	5
**2/64a**	524–531	32
**9/75**	528–535	76
**6/53**	544–551	4
**H62**	644–655	2

The results were confirmed in a reciprocal competition assay, using each mouse and rat monoclonal as a probe. Data are expressed as % of inhibition.

### Epitope Characterization: Binding Experiments on Point-Mutated HCV\E2 Clones

To define better the critical E2 residues involved in binding, the reactivity of Fab e20 to a panel of alanine point-mutated H77-derived HCV/E1-E2 (1a) clones [28] was determined. Five of these mutants (W420A, Y527A, W529A, G530A, D535A) completely inhibit HCV/E2 binding to CD81 also abrogating the infectivity of HCV pseudoparticles [28] . Differences in e20 binding to wild type H77 and to each mutant were determined by FACS, following the protocol described above. In this approach, the ALP98 mouse mAb (directed against a linear epitope outside the mutated regions) [29] was used to evaluate the expression level of the mutated E1E2.

### Neutralization Assays on HCV Pseudoparticles (HCVpp) and on HCV Cell Culture Infectious Viral Particles (HCVcc)

The neutralizing activity of Fab e20 was tested using a HCV pseudoparticle (HCVpp)-based neutralization assay, following already described protocols [26]. In particular, Fab e20 neutralizing activity was tested against pseudoparticles derived from murine leukemia virus (MLV) displaying unmodified and functional full-length E1-E2 proteins representative of all the major HCV genotypes. In brief, HEK293T cells were cotransfected with the MLV Gag-Pol packaging vector, the MLV-luciferase reporter gene, and the plasmid expressing HCV E1E2 from different genotypes. In all experiments, control particles expressing vesicular stomatitis virus (VSV) G protein were used. Two days after transfection, the medium containing HCVpp was collected, clarified, filtered through a 0.45-µm-pore-size membrane, and used for infection of Huh-7 cells. Three days following infection, the cells were analyzed on a luminometer using the Bright-Glo ™ luciferase assay kit (Promega). For all genotypes, but genotype 3 and genotype 6, the detected luminescence ranged from 10,000 to 20,000 relative luminescence units (RLU). For the neutralization assay, the HCVpp (10,000 RLU) were pre-incubated with different concentrations of Fab e20. The anti-HCV/NS3 Fab c33-3 was used as negative control.

The neutralizing activity of Fab e20 was also investigated using the cell culture infectious HCV (HCVcc) system, based on HCV genotype 2a strain JFH-1 [30], [31]. The infectivity of JFH-1 in the presence of different concentrations of Fab e20, and of the negative-control Fab (c33-3) was determined by quantitative reverse transcription-PCR, normalizing the viral RNA against the RNA of a housekeeping gene (glyceraldehyde-3-phosphate dehydrogenase)[32].

## Results and Discussion

The HCV genome encodes a mutation prone polymerase, resulting in the formation *in vivo* of a population of genetically related, but distinct variants. It is presently believed that the capacity of the virus to mutate contributes, together with the high replication rates, to the establishment of persistence. A hypervariable region (HVR1) of the HCV E2 glycoprotein has been observed to be a major determinant of isolate-specific neutralizing antibodies [33]. These antibodies provide, however, little protection against infection, due to the continuous sequence evolution of HVR1 [33]. Otherwise, it has been observed that broadly neutralizing antibodies are usually directed against highly conserved conformational epitopes of the E2 glycoprotein [21], [22], [24], [34], [35], [36], [37].

**Figure 1:**
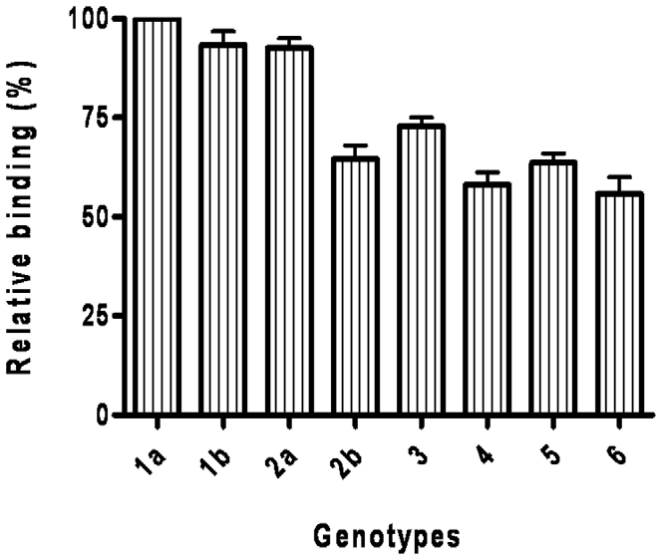
Binding activity of Fab e20 on E1E2 derived from different HCV genotypes. The following strains were used: genotype 1a (isolate H77.20); 1b (UKN1B12.16); 2a (UKN2A.2.4); 2b (UKN2B2.8); 3 (UKN3A1.28c); 4 (UKN4.21.16); 5 (UKN5.15.11) and 6 (UKN6.5.8). The broadly cross-reactive monoclonal AP33 was used to assess that the efficiency of transfection was comparable for all different genotypes. Binding activity for each genotype is expressed as percentage of the reactivity observed on E1E2 of genotype 1a (H77 strain). The means and standard errors of two replicate assays are reported.

Fab e20 investigated in this study was able to bind HCV/E2 derived from all tested genotypes (Figure 1), indicating the highly conserved nature of its epitope. The wide cross-reactivity of Fab e20 was also documented by immunoprecipitation of HCV/E2 on radiolabeled lysates from HEK293T cells expressing HCV surface proteins of different genotypes (data not shown). The broad cross-reactivity of Fab e20 prompted us to better characterize its epitope on HCV/E2. Previous data had already suggested that the epitope was conformationally constrained, as the Fab failed to bind either denatured HCV/E2 or multiple overlapping peptides encompassing the whole HCV/E2 sequence [23]. Moreover, Fab e20 did not show any reactivity in ELISA to five different Maltose Binding Protein-HCV/E2 fusion proteins ([E2a] - aa 384–444; [E2ab] - aa 384–512 [E2abc] - aa 384–583; [E2abcd] - aa 384–668 and [E2abcde] - aa 384–714) (Methods S1 and Table S1), or to five HVR multiple antigenic peptides [38] (MAP 291, MAP 313, MAP 442, MAP 455 and MAP 1013) (Methods S1 and Table S2), which were specifically recognized by some of the antibodies directed against linear epitopes used in this study. These data confirmed that e20 is directed against a conformational epitope which is retained only in the full length HCV/E2.

**Figure 2:**
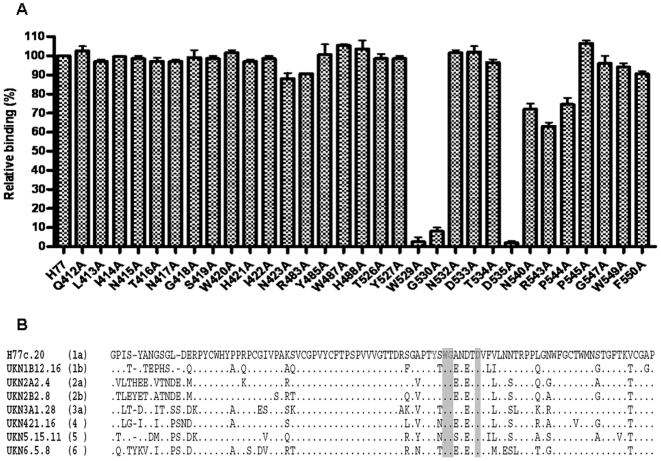
Residues recognized by Fab e20 on HCV/E2. (**a**) Reactivity of Fab e20 on a panel of mutated E1E2 glycoproteins derived from H77 strain. Binding activity, expressed as a percentage of the reactivity on wild type E1-E2 (H77 strain), is shown on the y axis; the mutated residues are shown on the x axis. Fab e20 was tested at 10 µg/ml. The means and standard errors of two independent assays are reported. (**b**) HCV/E2 partial sequence alignment of the HCV strains used in this study. The genotype of each strain is reported in brackets. Residues Y527, W529, G530 and D535 abrogating the binding to CD81 are depicted in red. Shaded grey boxes evidence the conserved residues recognized by e20.

In the competition assays, Fab e20 was strongly inhibited by 9/75, a rat mAb recognizing the linear epitope 528–535 on HCV/E2 (Table 1) [39]. Fab e20 also competed with rat mAb 1/39 (436–443) and, partially, with mouse mAb AP33 (412–423), the broadest cross-reactive and cross neutralizing antibody described to date (Table 1) [26], [40]. The competition with mAb 9/75 was confirmed by the fact that mutations at conserved positions 529, 530 and 535 on HCV/E2, which are located within the epitope recognized by mAb 9/75 and which are involved in the binding to CD81 [28], totally abrogated the binding of e20 (Figure 2a). These data show that these residues, highly conserved among different genotypes (Figure 2b), have a crucial role in generating the epitope recognized by Fab e20, and further indicate their importance in the production of anti-HCV/E2 broadly neutralizing antibodies [24], [26], [34], [36], [41]. On the contrary, in spite of the competition observed with AP33, mutations inside the AP33 epitope did not affect e20 binding, showing that the e20 conformational epitope does not contain the linear AP33 epitope, and that the observed competition may be mediated by steric hindrance, or by induced conformational changes of the antigen.

**Figure 3:**
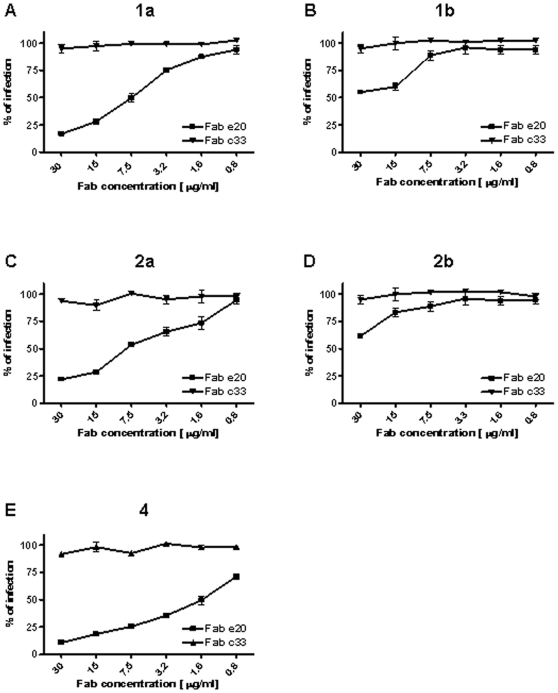
Neutralizing activity of Fab e20 using virus pseudoparticles (HCVpp). The following strains were used: genotype 1a UKN1A20.8 (**a**); E1E2 genotype 1b UKN1B5.23 (**b**); genotype 2a UKN2A1.2 (**c**); genotype 2b UKN2B1.1 (**d**); genotype 4 UKN4.21.16 (**e**). Data obtained from HCVpp infection in the presence of different concentrations of c33-3 (negative control) and e20 are presented as a percentage of the infection detected in the absence of antibody. The means and standard errors of two replicate assays are reported. HCVpp displaying genotype 3 (UKN3A1.28c) and genotype 6 (UKN6.5.8) did not give consistent signals in repeated assays, and were therefore not evaluated. No neutralizing activity was observed against genotype 5 (UKN5.15.11).

**Figure 4:**
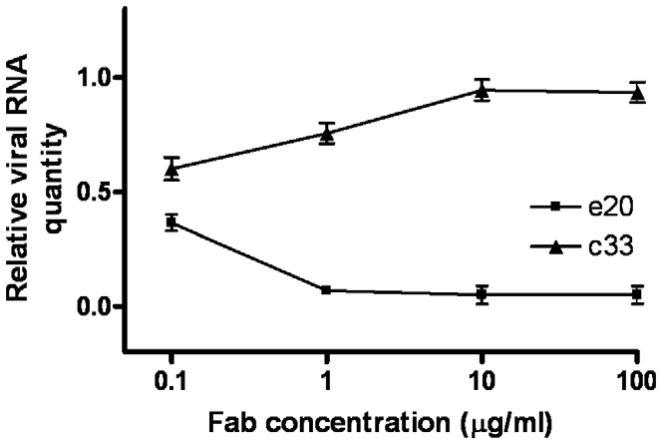
Neutralizing activity of Fab e20 using the HCVcc system (genotype 2a). The infectivity of JFH-1 in the presence of e20 and of negative-control Fab (c33-3) is presented as the viral RNA quantity normalized against glyceraldehyde-3-phosphate dehydrogenase RNA, as determined by quantitative reverse transcription-PCR [32]. The means and standard errors of three replicate assays are reported.

Fab e20 featured a potent neutralization activity against HCVpp derived from genotype 1a, with an IC50 (Fab concentration giving 50% neutralization) of 7.5 µg/ml (Figure 3a), and derived from genotypes 2a and 4, with IC50 values of 7.5 µg/ml and 1.6 µg/ml, respectively (Figure 3c and 3e). Finally, e20 was able to inhibit, albeit to a lesser extent, the infection of HCVpp 1b and 2b, with IC40 values of 15 µg/ml and 30 µg/ml, respectively (Fig. 3b and 3d). No neutralizing activity was observed against genotype 5 (UKN5.15.11). Unfortunately, it was not possible to perform the assay on genotypes 3 and 6, due to the very low and not reproducible infectivity observed for these genotypes [34]. Finally, the cross-neutralizing activity of e20 was also investigated using the cell culture infectious HCV (HCVcc) system based on HCV genotype 2a strain JFH-1 [30]. In this assay Fab e20 showed an even stronger neutralizing activity, since at a concentration as low as 1 µg/ml it was able to completely abrogate the infectivity of HCVcc (Figure 4).

Overall, the data presented here highlight that broadly cross-reactive and cross-neutralizing antibodies, as Fab e20, are generated during the natural course of HCV infection, and that they are directed against conserved epitopes on HCV/E2. We have observed that Fab e20 is able to bind all of the HCV/E2 genotypes tested, including the most phylogenetically distant genotype 2, and that it recognizes an epitope centred on three crucial residues (W529, D530 and G535). These residues, highly conserved among different HCV genotypes, are involved in E2 binding to the CD81, a possible co-receptor of HCV, and are recognized by other neutralizing antibodies [24], [34], [35], [37]. More in details, Fab e20 is one of the few human monoclonals described to date as capable of binding all major HCV genotypes. Three of these monoclonals (1∶7; A8 and CBH-5) share with e20 the residues D530 and G535 (1∶7 and A8 share also W529); however, although not easily comparable because tested in different settings, they feature different neutralizing activities against different genotypes, evidencing that other residues outside those studied may play a role [42], [43]. The main features of these broadly reacting antibodies, all tested as whole IgG1, are reported in Table 2 and are compared to the characteristics of e20, which has been tested as Fab fragment.

**Table 2:** Synopsis of published anti-HCV/E2 human monoclonals binding to all major HCV genotypes (1a, 1b, 2a, 2b, 3a, 4, 5, 6).

mAb	Crucial residues on HCV/E2 (H77 strain)	Neutralizing activity Assay format and genotype	Antibody format	References
e20	**W529**, **G530**,**D535**	7.5 HCVpp 1a	IgG1 Fab	This paper
		7.5 HCVpp 2a		
		1.6 HCVpp 4		
		<<1 HCVcc 2a		
1∶7	G523, **W529**, **G530**, **D535**	0.06 HCVcc 2a	whole IgG1	[35]
A8	G523, **W529**, **G530**, **D535**	0.56 HCVcc 2a	whole IgG1	[35]
CBH-5	C494, V497, G523, P525, **G530**, **D535**, N540, R614, H617, Y618, P619, T621, F624	1.77 HCVpp 1b	whole IgG1	[37], [42], [43], [50], [51]
		0.1 HCVpp 2a		
		13 HCVpp 2b		
		0.056 HCVcc 2a		
		0.04 HCVcc 2b		
CBH-7	C494, V497, N540, W549, R614, H617, Y618, P619, T621, F624	25.58 HCVcc 2a	whole IgG1	[37], [42], [43], [50], [51]
		1.3 HCVcc 2b		

The E2 residues involved in the binding of each antibody are reported (those crucial for e20 binding are in bold and underlined). Only the concentrations giving 50% neutralization (IC50) against HCV pseudoparticles (HCVpp) derived from different genotypes, or against HCV cell culture (HCVcc) strains, are reported. Punctual IC50 values are not available for mAbs 1∶7 and A8 against HCVpp [35], and for CBH-5 and CBH-7 against HCVpp derived from genotypes 1a, 3a, 4, 5 and 6 [37], [42], [43], [50], [51]. E20 is the only monoclonal tested as Fab fragment.

An intriguing aspect is the fact that Fab e20, although recognizing genotype 5 E2 on transfected cells, was not able to neutralize in our hands HCVpp obtained with the same glycoprotein. In theory, this can be due (i) to low sensitivity of the HCVpp neutralization assay [37] or, (ii) to the role played by residues located outside the CD81 binding site and not tested in the mutants panel, but possibly important for the neutralizing activity of e20 against genotype 5 [44].

Finally, it is important to note that e20 biological activity was tested using the antibody as a Fab fragment, and that its activity may increase in the whole immunoglobulin format [45]. That may result in increased neutralizing potency not only to genotypes 1a, 2a and 4, but also to genotypes 1b and 2b. Moreover, the better knowledge of the broadly neutralizing epitopes on HCV/E2 glycoprotein, and the availability of a panel of antibodies with broadly neutralizing characteristics, similar to those observed for e20, may also allow the use of a balanced combination of neutralizing antibodies, thus resulting in a synergic enhancement of the overall neutralizing activity (as already observed with HIV) [46], [47], [48], [49].

## Supporting Information

Table S1 Binding of e20 Fab to maltose binding protein-E2 fusion constructs (O.D.450). The human Fab C33 directed against HCV/NS3 was used as negative control; mouse Mabs 7/59, 7/16b and 6/53 were used as positive controls.
(0.03 MB DOC)

Table S2 Binding of human anti-HCV/E2 Fab e20 to multiple antigenic peptides (MAP) (O.D.450). The sequence of each MAP is reported. The human Fab C33 was used as negative control; mouse Mabs 7/59 and 9/27 were used as positive controls.
(0.03 MB DOC)

Methods S1 Binding of human anti-HCV/E2 Fab e20 to Maltose Binding Protein (MBP)-HCV/E2 fusion proteins. Binding of human anti-HCV/E2 Fab e20 to Hypervariable region 1 (HVR1) multiple antigenic peptides.
(0.02 MB DOC)
