# Supplementary material for: Hepatitis C Virus (HCV) Infection May Elicit Neutralizing Antibodies Targeting Epitopes Conserved in All Viral Genotypes
Source: PLoS One. 2009 Dec 11;4(12):e8254. doi: 10.1371/journal.pone.0008254 (PMC2785886; doi:10.1371/journal.pone.0008254)
Supplement: Table S1 — Binding of e20 Fab to maltose binding protein-E2 fusion constructs (O.D.450). The human Fab C33 directed against HCV/NS3 was used as negative control; mouse Mabs 7/59, 7/16b and 6/53 were used as positive controls. (0.03 MB DOC) [file pone.0008254.s001.doc]

**Table S1**

| Antibody | MBP-E2 fusion protein | | | | | |
| --- | --- | --- | --- | --- | --- | --- |
|  | **E2-a** | **E2-ab** | **E2-abc** | **E2-abcd** | **E2-abcde** | **MBP** |
| **e20** | 0.05 | 0.06 | 0.05 | 0.03 | 0.04 | 0.05 |
| **C33 (neg)** | 0.05 | 0.05 | 0.07 | 0.03 | 0.03 | 0.03 |
| **7/59** | 1.80 | 1.78 | 1.90 | 1.85 | 1.90 | 0.06 |
| **7/16b** | 0.05 | 1.98 | 1.78 | 1.80 | 1.85 | 0.04 |
| **6/53** | 0.04 | 0.03 | 1.94 | 1.85 | 1.90 | 0.05 |
