# Supplementary material for: Hepatitis C Virus (HCV) Infection May Elicit Neutralizing Antibodies Targeting Epitopes Conserved in All Viral Genotypes
Source: PLoS One. 2009 Dec 11;4(12):e8254. doi: 10.1371/journal.pone.0008254 (PMC2785886; doi:10.1371/journal.pone.0008254)
Supplement: Table S2 — Binding of human anti-HCV/E2 Fab e20 to multiple antigenic peptides (MAP) (O.D.450). The sequence of each MAP is reported. The human Fab C33 was used as negative control; mouse Mabs 7/59 and 9/27 were used as positive controls. (0.03 MB DOC) [file pone.0008254.s002.doc]

**Table S2**

**MAP 1013 (strain H 77)** ETHVTGGSAGHTVSGFVSLLAPGAKQN

**MAP 455**  QHTTTGGQAGHQAHSLTGLFSPGAKQN

**MAP 313** TTTTTGGVQGHTTRGLVRLFSLGSKQN

**MAP 442** QTHTTGGVVSHQTRSLVGLFSPGPQQN

**MAP 291** ETHSVGGSAAHTTSRFTSLFSPGPQQN

| Antibody | Multiple antigenic peptide | | | | | |
| --- | --- | --- | --- | --- | --- | --- |
|  | **1013 (Strain H)** | **455** | **442** | **313** | **291** | **Negative**  **control** |
| **e20** | 0.06 | 0.05 | 0.04 | 0.08 | 0.08 | 0.06 |
| **C33 (neg)** | 0.04 | 0.07 | 0.04 | 0.06 | 0.04 | 0.06 |
| **mouse 7/59** | 1.86 | 1.80 | 0.23 | 0.15 | 0.13 | 0.08 |
| **mouse 9/27** | 1.86 | 0.24 | 0.12 | 0.13 | 0.12 | 0.09 |
