# Supplementary material for: Hepatitis C Virus (HCV) Infection May Elicit Neutralizing Antibodies Targeting Epitopes Conserved in All Viral Genotypes
Source: PLoS One. 2009 Dec 11;4(12):e8254. doi: 10.1371/journal.pone.0008254 (PMC2785886; doi:10.1371/journal.pone.0008254)
Supplement: Methods S1 — Binding of human anti-HCV/E2 Fab e20 to Maltose Binding Protein (MBP)-HCV/E2 fusion proteins. Binding of human anti-HCV/E2 Fab e20 to Hypervariable region 1 (HVR1) multiple antigenic peptides. (0.02 MB DOC) [file pone.0008254.s003.doc]

**Binding of human anti-HCV/E2 Fab e20 to Maltose Binding Protein (MBP)-HCV/E2 fusion proteins.**

Fragments encoding portions of HCV envelope glycoprotein E2 Strain H77 were generated by PCR and inserted into the pMal-C2 vector (New England Biolabs, USA). The following constructs were generated expressing regions of E2: E2a - aa 1-61 (384-445 on HCV polyprotein); E2ab - aa 1-129 (384-513); E2abc - aa 1-200 (384-584); E2abcd - aa 1-285 (384-669) and E2abcde - aa 1-331 (384-715). Parental pMal and fusion protein constructs were transformed into DH5a cells (GIBCO/BRL/Life Sciences, USA), cultures induced and the proteins purified by amylose column chromatography (New England Biolabs, USA) as per manufacturer’s instructions.

The different fusion proteins (4 µg/ml) were used to coat ELISA plates (Costar) overnight at 4°C. The plates were the washed and blocked with PBS/1%BSA for 1h at 37°C, 50 µl of appropriate dilution of Fab preparation in PBS/1%BSA was added and incubated for 2h at 37°C. Fab e20 and the mouse control mAbs were tested at 10ug/ml. The plates were washed 10 times with phosphate buffer saline (PBS)/0.05% Tween-20 (Sigma) and 50µl of a 1:700 dilution in PBS of horseradish peroxidase conjugate goat anti-human Fab (Sigma) were added. In the case of mouse control mAbs an anti-mouse Fc conjugate (Sigma) was used and in the case of human sera anti-human Fc conjugate was employed. After 2h at 37°C plates were washed as above, 100µl of substrate (Sigma) were added and plates were read for OD at 450nm after 30 min at room temperature in the dark. Data presented are the average of a minimum of four readings. All assays were performed at least in double. A negative control antigen (BSA) was always included and the OD reading was subtracted as background.

Binding of human anti-HCV/E2 Fab e20 to Hypervariable region 1 (HVR1) multiple antigenic peptides.

HVR1 multiple antigenic peptides (MAPs) were kindly provided by A.Nicosia (IRBM, Pomezia, Italy). Four HVR1 sequences (MAP 455, MAP 313, MAP 442 and MAP 291 - for sequences see Supplementary table 2) and strain H77 HVR1 (1013 - ETHVTGGSAGHTVSGFVSLLAPGAKQN) were synthesized as multiple antigenic peptides (MAP). These HVR1 MAPs alongside an unrelated MAP were coated directly onto Immulon II EIA plates (Dynal, UK) at 1 µg/ml overnight at 4°C. ELISA was performed as described above
